# What hybrids reveal about the notion of species in *Leishmania*

**DOI:** 10.1371/journal.ppat.1014468

**Published:** 2026-08-03

**Authors:** Marcela Fuentes-Carias, Viviane Noll Louzada-Flores, Isabelle Louradour

**Affiliations:** Institut Pasteur, Université Paris Cité, INSERM U1347, Unité de Parasitologie moléculaire et Signalisation, Paris, France; Institute of Parasitology, Biology Centre, Czech Academy of Sciences, CZECHIA

## *Leishmania*: Many hosts, vectors, clinical forms … and “species”?

Parasites of the genus *Leishmania* are the causative agents of the neglected tropical diseases known collectively as leishmaniases. These parasites present a digenetic life cycle alternating between vertebrate and sand fly insect hosts, including *Phlebotomus* in the Old World and *Lutzomyia* in the New World. *Leishmania*’s transmission cycle can be zoonotic, with dogs, rodents and other wild or domestic mammals acting as reservoirs, or anthroponotic, where humans are the main reservoir. More than 50 *Leishmania* species have been described, among which at least 20 are pathogenic to humans, displaying a wide variation of host range, vector associations and clinical outcomes [1].

Clinically, leishmaniases range from localized skin disease to fatal systemic infection, broadly grouped into cutaneous leishmaniasis (CL)**,** mucocutaneous leishmaniasis (MCL) and visceral leishmaniasis (VL). Additional forms include diffuse or disseminated CL and post-kala-azar dermal leishmaniasis (PKDL), a skin pathology emerging in treated VL patients [1]. CL, the most common form, typically presents nodular or ulcerative skin lesions, whereas MCL affects the nasal and oropharyngeal tracts. VL affects the spleen, liver, and bone marrow, and is fatal if untreated.

Although *Leishmania* species are associated with specific clinical manifestations (e.g., *L. donovani* and *L. infantum* with VL; *L. tropica*, *L. major*, *L. braziliensis,* and others with CL/MCL), an increasing number of parasites causing atypical clinical and epidemiological patterns have been reported. Emblematic examples include Turkish *L. infantum* parasites causing exclusively CL [2], Indian *L. donovani* isolates responsible for unusual cutaneous disease [3] as well as Italian *L. infantum* parasites causing severe CL or VL [4]. Isolates from Sri Lankan CL patients perfectly illustrate this complexity, as both *L. donovani* and *L. tropica* have been reported as causing CL in the region, with the *L. tropica* isolates exhibiting evidence of ancient hybridization events, i.e., genetic exchange between distinct *Leishmania* strains [5]. The very possibility of hybridization between different *Leishmania* parasites, sometimes separated by substantial genetic distances, lies at the heart of the central question addressed here: do bona fide *Leishmania* species truly exist, or is the very notion of “species” poorly suited for these microorganisms?

## What is a *Leishmania* species?

The species concept, first theorized by Ernst Mayr, is traditionally defined as a group of organisms that can interbreed and produce fertile offspring while being reproductively isolated from other groups, or more broadly, as populations that are genetically and phenotypically cohesive and distinct from others [6]. Historically, *Leishmania* “species” were classified mainly according to clinical presentation and tropism, with categories reflecting predominant symptoms in patients rather than underlying genetic relationships. The advent of genome-wide analyses has revealed that this symptom-based taxonomy is often inaccurate: parasites causing distinct clinical outcomes can belong to the same genetic clade, while closely related lineages may display markedly different disease manifestations [7].

*L. infantum* provides a clear example: although primarily associated with VL worldwide, it also frequently causes CL, and even rare mucosal cases- all within the same genetic species complex as confirmed by multilocus genotyping and whole-genome sequencing. Conversely, *L. braziliensis* causes CL/MCL but can occasionally produce visceral symptoms within the same genetic group [8]. These discrepancies highlight how clinical outcomes do not always align with species boundaries. It is equally important to note, however, that disease manifestation is not driven only by the parasite, but is also strongly influenced by the host’s genetic background and immune status [9].

## *Leishmania* species can meet … and mate

The rapid advancement of sequencing technologies has revealed numerous natural *Leishmania* hybrids over recent decades, including intraspecific and interspecific hybrids resulting from crosses between genetically distant species [10]*.* As sequencing coverage increases across regions and hosts, new hybrid genotypes continue to be detected, indicating that recombination is more recurrent than initially appreciated and further challenging the robustness of conventional species boundaries in *Leishmania*.

**Fig 1:**
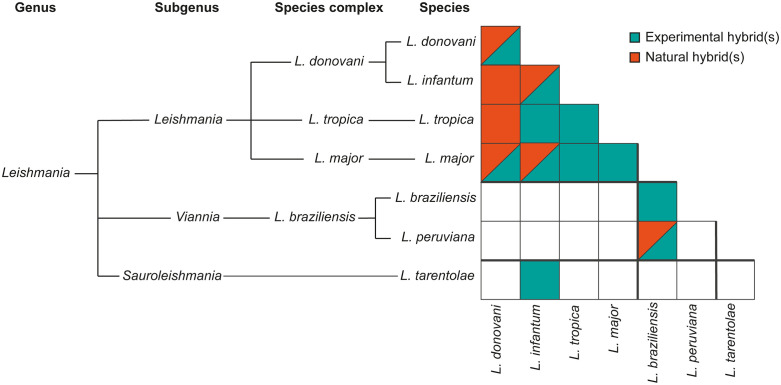
Overview of natural and experimental hybrids reported to date. A variety of natural intra- and interspecific hybrids have been identified over the past decades. Experimentally, different combinations of *Leishmania* species have been tested to generate hybrids, with variable success, either through sand fly co-infections or *in vitro* hybridization. For a comprehensive list of reported *Leishmania* hybrids, see [10].

Hybrid formation has also been achieved experimentally. *In vivo* hybrids are generated by co-infecting sand flies with two parental strains, followed by double-drug selection on gut extracts. This approach has yielded both intra- and interspecific hybrids, including “selfies”, i.e., sexual progeny from individuals deriving from the same clone [10,11]. Hybrids can also be generated *in vitro*, as exemplified by an “extreme” cross between *L. infantum* and *L. tarentolae*, two parasites from different phylogenetic subgenera [12]. Experimentally generated hybrids can be transmitted and stably maintained, demonstrating genomic compatibility even between evolutionarily distant species and confirming that these products are not evolutionary dead-ends. The frequency of successful hybridization varies widely across parental combinations, with some crosses failing entirely, suggesting that compatibility determinants remain to be discovered (Fig 1). Collectively, these findings suggest an absence of mating barrier between the so-called *Leishmania* species.

## How do *Leishmania* hybrids form?

Whole-genome sequencing of experimental hybrids has revealed Mendelian inheritance of nuclear markers, and *in vivo* backcrosses have shown evidence of meiosis-like recombination and chromosome segregation [13], also supported by the observation of phased haplotypes and non-random inheritance of heterozygous genomic blocks in natural populations [14]. Despite these advances, the cellular and molecular mechanisms underlying hybrid formation remain poorly understood. The parasite stages that undergo fusion have not been identified, and the nature of the fusion and meiotic machinery remains largely unknown. Most natural hybrids, like their presumed parental “non-hybrid” strains, are diploid, although polyploid forms have occasionally been reported. In contrast, experimental hybrids, particularly those generated *in vitro*, frequently display triploid or tetraploid karyotypes [15,16]. As haploid gametes have never been observed in *Leishmania*, the frequent recovery of experimental polyploid hybrids may indicate that *Leishmania*’s meiotic-like cycle starts from a diploid–diploid fusion process later eventually followed by a meiotic reduction, or reflect that *in vitro* hybridization result from abnormal fusion events rather than from the meiosis-like cycle naturally occurring during transmission in sand flies.

Hybridization events are typically rare, and no standardized protocol currently allows mating to be induced on demand, which continues to hamper mechanistic studies. Several triggering factors have been identified: stress conditions such as DNA damage can markedly increase hybrid yields *in vitro*, and the presence of IgM in the sand fly blood meal has been shown to promote hybrid formation by inducing parasite aggregation [15,17]. Several molecular actors have also been implicated, including the fusogens HAP2 and the meiosis-related enzyme HOP1, yet the hybridization process remains only partly described [10,15,18]. While *Leishmania* is capable of a meiosis-like sexual cycle in the sand fly vector, and under certain conditions *in vitro*, the key cellular players, molecular machinery and regulatory triggers remain largely enigmatic.

## Genome plasticity stretches what we call a species

*Leishmania* parasites are exceptional in their genome plasticity, i.e., their capacity to repeatedly and reversibly reshape genome structure to modulate gene dosage and adapt to environmental pressures [19]. Widespread aneuploidy (gain or loss of entire chromosomes) and large-scale copy number variations (CNVs) are frequent and well tolerated, and may reflect adaptation to distinct ecological niches. Single-cell genomic studies have revealed that mosaic aneuploidy—in which multiple karyotypes coexist within a single clonal population—can emerge rapidly, particularly under environmental stress [20]. Beyond copy number changes, *Leishmania* genomes harbor a broad spectrum of structural variation, including translocations, large-scale rearrangements, loss of heterozygosity, telomeric amplifications, and tandem gene array expansions. Interspecific differences in chromosome number further illustrate this fluidity: for instance, the *L. mexicana* complex carries 34 chromosomes, compared to 36 in *L. infantum* and *L. donovani*, a discrepancy attributed to chromosomal fusions that nonetheless preserve overall gene content.

**Fig 2:**
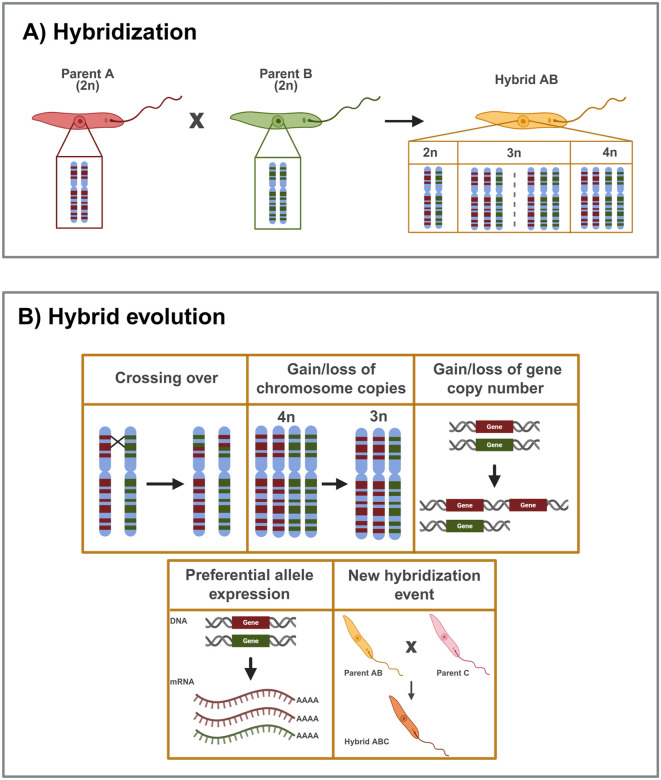
Formation and evolution of *Leishmania* hybrids. **A)** Hybrids arise from two parental diploid strains through a cryptic, facultative sexual cycle involving a meiosis-like process. While most hybrids are diploid, triploid or tetraploid forms can occur. **B)** Once formed, hybrids are subject to evolutionary pressures that reshape their genomes through crossing over, aneuploidies, and CNVs. Hybrid genomes may also display preferential allele expression, and hybrids can participate in further hybridization or backcrossing with parental strains. Created in BioRender. Fuentes Carias, M. (2026) https://BioRender.com/xamb9vl.

The facultative sexual cycle represents perhaps the most extreme manifestation of this genomic plasticity, generating hybrid genomes subsequently sculpted by evolutionary forces (Fig 2). At the gene level, hybrid fitness can be further modulated by preferential expression of one parental allele, extending plasticity to post-transcriptional and post-translational regulation [12]. Interestingly, hybrids can present a higher fitness than each parent. For instance, by expressing *L. major* surface molecules (namely lipophosphoglyan or LPG), *L. major/L. infantum* hybrids are able to develop in sand flies that normally would support only *L. major*, thus widening hybrids transmission potential [21]. Despite this remarkable fluidity, population genomic surveys across hundreds of isolates consistently reveal well-supported genetic clusters broadly corresponding to classical species or species complexes, with clear geographic and ecological structuring and limited evidence of recent interspecific recombination [22]. These findings affirm the practical utility of current species boundaries for taxonomy and epidemiological surveillance. However, recent population genomics suggests genetic exchange is far more common than this clustering implies, since in over 70% of examined Old World isolates, the high heterozygosity can be separated into haplotypes inherited from two different parents, showing mixed ancestry within and between species [14]. Several recognized *Leishmania* species may therefore be better seen as parts of broader species complexes than as separate, reproductively isolated groups. Yet, hybridization also carries epidemiological weight since hybrids can seed new foci of leishmaniasis, and the progeny of a single hybridization event may colonize a relatively large geographic area [5]. In certain groups, including the *L. braziliensis* complex, *L. tropica*, *L. aethiopica*, and the *L. donovani* complex, frequent hybridization, pervasive aneuploidy, CNV accumulation, cellular mosaicism, and ecological divergence collectively erode the strict biological species concept based on reproductive isolation. These taxa may be more appropriately conceived as subspecies or ecotypes within broader evolutionary complexes. More broadly, *Leishmania* taxonomy likely spans a range of evolutionary relationships rather than a fixed set of rigid species since some taxa hybridize readily, while others seem reproductively isolated. Whether that isolation is truly biological or simply reflects geographic, vector, or ecological separation is still unclear, especially as growing evidence shows that overlapping transmission cycles favor hybridization.

To conclude, *Leishmania* has long been treated as a set of discrete, well-separated species, but whole-genome sequencing has revealed this view to be inadequate, given the extensive natural hybridization. Genetic species boundaries largely break down, revealing instead genetically structured lineages with remarkable capacity for gene flow and genome remodeling. Rather than fixed genomic entities, *Leishmania* “species” are better conceptualized as dynamic clouds of karyotypes and copy-number variation profiles, continually reshaped by evolutionary and ecological pressures.
